# Enhancing postpartum cardiometabolic health for women with previous gestational diabetes: Next steps and unanswered questions for pharmacological and lifestyle strategies

**DOI:** 10.1111/dom.16070

**Published:** 2024-11-18

**Authors:** Rajna Golubic, Josip Car, Kypros Nicolaides

**Affiliations:** ^1^ Diabetes Trials Unit, Oxford Centre for Diabetes, Endocrinology and Metabolism, Radcliffe Department of Medicine University of Oxford Oxford UK; ^2^ School of Life Course and Population Sciences, King's College London London UK; ^3^ The Fetal Medicine Research Institute London UK

**Keywords:** dietary intervention, exercise intervention, gestational diabetes, incretin therapy, obesity care, weight management

Gestational diabetes mellitus (GDM) affects 14% of pregnancies worldwide, and its prevalence is on the rise.^1^ This increase is driven by multiple factors: the growing rates of obesity and overweight, advanced maternal age, enhanced detection through improved screening methods and diagnostic criteria, shifts in diet, physical activity and lifestyle, as well as ethnic and genetic influences. GDM is associated with a 10‐fold increased risk of developing type 2 diabetes (T2D) compared with a normoglycaemic pregnancy, particularly within the first 5 years after childbirth. Insulin resistance appears to be the predominant mechanism.^2^ Women with previous GDM are also at a 2.4‐fold increased risk of metabolic syndrome^3^ and twofold increased risk of cardiovascular disease (CVD).^4^ Studies focusing on long‐term cardiovascular outcomes in women with a history of GDM demonstrated that the elevated risk of CVD is independent of developing T2D and is most prominent in the first 10 years after pregnancy.^4^ Lifestyle modifications targeting weight loss, diet and exercise have been recommended to reduce the risk of T2D postpartum.^5^ However, adherence to lifestyle modification is low.^6^, ^7^ Therefore, pharmacologic interventions have been explored for reducing cardiometabolic risk postpartum.

Several pharmacologic agents^8^ were investigated in overweight and obese women with past GDM focusing on preventing T2D (metformin, pioglitazone, troglitazone) or on the effects on cardiometabolic risk factors (dapagliflozin, liraglutide) and showed promising results. Metformin,^9^ pioglitazone^10^ and troglitazone^11^ demonstrated up to 55% reduction in the risk of T2D compared with the standard of care over the period of 3 months to 3 years. However, troglitazone was withdrawn from the market in 2000 due to hepatotoxicity. Dapagliflozin and metformin were more effective in reducing fasting plasma glucose and glucose excursions and improving lipid profile versus metformin or dapagliflozin monotherapy after 24 weeks of treatment.^12^ Liraglutide and metformin combined resulted in a greater reduction in weight, central adiposity and improvement in insulin sensitivity versus metformin alone after 84 weeks of treatment.^13^ However, liraglutide and metformin commonly cause gastrointestinal discomfort, nausea or vomiting, which may lead to reduced adherence. Furthermore, the populations studied were predominantly White and low socio‐economic strata were not represented, indicating that this gap needs to be addressed in future research. No other pharmacotherapies have been studied in the context of preventing T2D after GDM^8^ and long‐term effects are unknown.

Incretin‐based therapies including liraglutide, semaglutide (GLP‐1 receptor agonists) and tirzepatide (GLP‐1/GIP agonist) have been licensed for managing T2D and obesity without diabetes alongside lifestyle modification.^14^, ^15^, ^16^ Weight lowering effects of these medications are achieved via a centrally mediated decrease in appetite and by delaying gastric emptying.^17^ In people living with obesity and without diabetes, mean weight loss associated with these therapies versus placebo was 5.6 kg over 56 weeks for liraglutide,^18^ 12.7 kg over 68 months for semaglutide^19^ and 16.1–23.6 kg over 72 weeks for tirzepatide. Therefore, in the United Kingdom, liraglutide and semaglutide have been approved for treating obesity without diabetes while the technology appraisal from National Institute for Health and Care Excellence is expected by the end of 2024. Although effective in reducing body weight and fat mass, glucagon‐like‐peptide‐1 (GLP‐1) based treatments cause a reduction in lean body mass by 25%–39%, which is an independent predictor of poor health outcomes and emphasizes the need for concurrent dietary and exercise interventions to prevent muscle loss.^20^ With multiple favourable pleiotropic effects on cardiometabolic health, which go beyond glucose and weight lowering,^21^ these therapies may be beneficial for overweight and obese women with previous GDM to reduce interpregnancy weight gain, or achieve desirable weight loss and metabolically optimize them for the following pregnancy, thus decreasing the risk of future GDM and T2D. However, there is uncertainty as to whether these therapies can be used in the postpartum period. The optimal timing of initiating pharmacotherapy in combination with lifestyle modification postpartum and duration of such interventions to achieve the best cardiometabolic outcomes is unknown. Long‐term efficacy and safety of pharmacotherapies to prevent T2D and CVD and reduce intermediate risk factors (dyslipidaemia, hypertension, prediabetes) in this population are not known and larger trials of incretin‐based and other therapies are needed to build the evidence base and support clinical guidance considering their young age and maternal and foetal outcomes of possible future pregnancies.

Pharmacological interventions for diabetes prevention should always be combined with lifestyle modifications. The efficacy of lifestyle interventions to prevent or delay T2D in high‐risk groups has been demonstrated in clinical trials.^22^, ^23^, ^24^, ^25^ Diabetes Prevention Program showed that in people with elevated fasting plasma glucose or in those with impaired glucose tolerance 150 min/week of physical activity reduced the incidence of T2D by 58% versus placebo and metformin reduced this risk by 31% versus placebo during 2.8 years.^22^ Da Quing Study demonstrated that in people with impaired glucose tolerance, lifestyle interventions over 6 years can prevent or delay T2D by up to 14 years after the active intervention versus control.^23^ Finnish Diabetes Prevention Study reported that in people with impaired glucose tolerance, lifestyle intervention (diet and exercise) reduced T2D risk by 58% versus control over 3.2 years.^24^ The National Health Service Diabetes Prevention Programme (NHSDPP) is an interventional programme implemented at scale in England aiming to prevent T2D in adults at risk (HbA1c 42–47 mmol/mol or fasting plasma glucose 5.5–6.9 mmol/L) through behaviour change achieved via health coaching and group support.^26^ NHSDPP has been more effective than usual care in reducing T2D risk with evidence of cost‐effectiveness, but the uptake was ~50%.^26^ Main strategies proposed to implement and scale interventions while ensuring fidelity (i.e. the extent to which the intervention is implemented as intended) include providing a theoretical underpinning of the programme (logic model), involving professionals trained in behavioural change techniques, using clear criteria to evaluate intervention providers, robust quality assurance and investigating whether particular parts of the intervention or formats of delivery are better received by specific populations.^27^ While strategies to prevent T2D targeting at‐risk groups and whole populations have been cost‐effective or cost‐saving,^28^ a systematic review of cost‐effectiveness of interventions to improve cardiometabolic outcomes in women with previous GDM found no randomized controlled trials reporting cost‐effectiveness of lifestyle or pharmacologic interventions and concluded that rigorously designed large‐scale trials are needed to address this gap.^29^

Additional unanswered questions concern the impact of combined interventions on foetal outcomes in subsequent pregnancies as well as on psychological well‐being, quality of life, breastfeeding outcomes and maternal–infant bonding. There is a lack of evidence of the role of digital health technology in enhancing the effectiveness of and adherence to combined pharmacotherapy and lifestyle interventions in women with previous GDM calling for more research in this area. Little is known about the influence of genetic factors on the effectiveness of combined pharmacotherapy and lifestyle interventions in women with a history of GDM.

Future studies must address lack of diversity by including non‐White women from low socio‐economic backgrounds. They should also explore strategies to overcome the barriers to implement behaviour change with or without pharmacotherapy across various healthcare settings including those with limited resources and examine how cultural beliefs and practices influence the acceptance of and adherence to these interventions in diverse populations. These studies should adopt a multi‐disciplinary approach integrating expertise in women's health, diabetes and cardiometabolic disorders and behaviour change. Finally, current guidelines are limited by a one‐size‐fits‐all approach, offering annual HbA1c monitoring and generic advice on lifestyle modifications aimed at weight control, diet and exercise without providing specific/personalized guidance.^5^

In summary, future studies involving women with past GDM should prioritize addressing the unresolved questions regarding the efficacy, safety (short‐ and long‐term) and cost‐effectiveness of postpartum interventions that combine pharmacotherapies with lifestyle modification, compared with lifestyle modification alone in relation to cardiometabolic health. Future research in women with a history of GDM should focus on generating evidence to support personalized postpartum advice on diet, exercise and other components of lifestyle (sleep, stress management) with or without pharmacotherapy tailored to each woman's cardiometabolic risk and potential benefit of each intervention component.

## CONFLICT OF INTEREST STATEMENT

All authors declare no conflict of interest.

### PEER REVIEW

The peer review history for this article is available at https://www.webofscience.com/api/gateway/wos/peer‐review/10.1111/dom.16070.

## Data Availability

Data sharing is not applicable to this article as no new data were created or analysed in this study.
